# Aortic Valve Annular Properties in Cardiac Amyloidosis—Insights from the Three-Dimensional Speckle Tracking Echocardiographic MAGYAR-Path Study

**DOI:** 10.3390/biomedicines14020488

**Published:** 2026-02-23

**Authors:** Attila Nemes, Nóra Ambrus, Zita Borbényi

**Affiliations:** Department of Medicine, Albert Szent-Györgyi Medical School, University of Szeged, H-6725 Szeged, Hungary; ambrusnora@gmail.com (N.A.); borbenyi.zita@med.u-szeged.hu (Z.B.)

**Keywords:** echocardiography, left ventricular, cardiac amyloidosis, aortic valve annulus, three-dimensional

## Abstract

**Introduction.** The etiology of cardiac amyloidosis (CA) involves the systemic or localized deposition of misfolded amyloid proteins within the myocardial interstitium and valvular structures. The primary objective of this study was to employ three-dimensional speckle-tracking echocardiography (3DSTE) to perform a detailed analysis of the aortic valve annulus (AVA) and left ventricular (LV) strains in CA patients and to compare these parameters with those of matched healthy controls. **Methods.** The initial cohort for this study comprised 35 individuals diagnosed with CA. However, 12 patients were subsequently excluded from the final analysis due to suboptimal image quality precluding accurate measurement of AVA dimensions and/or LV strains. The final analytical group, therefore, consisted of 23 CA patients (14 males), with a mean age of 64.6 ± 7.1 years. The results obtained from the CA patient group were compared with those of a healthy control cohort comprising 23 individuals (14 males; mean age: 53.2 ± 5.3 years). **Results.** In CA patients, AVA area was greater in end-diastole in 11 out of 23 cases (48%), and in end-systole in 8 out of 23 cases (35%), while it proved to be equal in 4 out of 23 cases (17%). The ratio of healthy controls with greater end-diastolic AVA area (12 out of 23, 52%) and greater end-systolic AVA area (11 out of 23, 48%) did not differ from that of CA patients. End-diastolic and end-systolic maximum and minimum AVA diameters, areas and perimeters did not differ between CA patients and matched controls. AVA plane systolic excursion (AAPSE) was found to be significantly impaired in all CA patients irrespective of AVA area size. Basal LV radial (RS), circumferential (CS) and longitudinal (LS) strains were reduced in CA patients compared with those of controls. End-systolic AVA dimensions tended to be reduced in CA patients with greater end-diastolic AVA area compared with those with greater end-systolic AVA area. While basal LV-RS and LV-CS proved to be similar between CA subgroups, basal LV-LS tended to be higher in CA patients with greater end-systolic AVA area. Controls with greater end-diastolic AVA area showed lower basal LV-RS and LV-LS compared with those with greater end-systolic AVA area. CA patients with equal end-diastolic and end-systolic AVA area (*n* = 4) showed similarly reduced AAPSE, basal LV-RS, basal LV-CS and LV-LS. **Conclusions.** In the presence of CA, the AVA is not dilated; however, its spatial displacement is reduced, suggesting its functional impairment, as represented by AAPSE, possibly due to the reduction in all concomitant LV strain parameters.

## 1. Introduction

The etiology of cardiac amyloidosis (CA) involves the systemic or localized deposition of misfolded amyloid proteins within the myocardial interstitium and valvular structures [1,2,3,4,5,6,7,8]. In clinical practice, diagnosing CA remains extremely challenging, and the 2021 European Society of Cardiology (ESC) consensus document provides a robust diagnostic framework for clinicians [8]. Although there is extensive literature on the myocardial, valvular, and vascular abnormalities associated with CA, the aortic valve (AV) and its annulus (AVA) remain less well-studied entities [1]. Three-dimensional speckle-tracking echocardiography (3DSTE) may help address this limitation, as it is considered to be a sophisticated tool that enables concurrent assessment of AVA dimensions and regional left ventricular (LV) function (measured as myocardial strains) within a comprehensive 3D framework [9,10]. Therefore, the primary objective of this study was to employ 3DSTE to perform a detailed analysis of the AVA and LV strains in CA patients and to compare these parameters with those of matched healthy controls.

## 2. Methods

### 2.1. Patient Population

The initial cohort for this study comprised 35 individuals diagnosed with CA. However, 12 patients were subsequently excluded from the final analysis due to suboptimal image quality precluding accurate measurement of AVA dimensions and/or LV strains. The final analytical group, therefore, consisted of 23 CA patients (14 males), with a mean age of 64.6 ± 7.1 years. For the diagnosis of CA, international guidelines were followed [8]. Biopsy was performed in all patients to confirm the diagnosis of CA, identifying light chain (AL) amyloidosis in 20 cases and transthyretin (TTR) amyloidosis in 3 cases. The results obtained from the CA patient group were compared against a healthy control cohort comprising 23 individuals (14 males; mean age: 53.2 ± 5.3 years). Controls were specifically recruited to be age- and gender-matched to the patient group and exhibited no known disorders or pathological conditions that might confound the study findings. According to the literature, at the population level, approximately 60% of (healthy) subjects show greater end-systolic AVA area and approximately 30% have greater end-diastolic AVA-A [11,12]. During the selection of healthy control subjects, particular attention was paid to ensuring that this ratio was similar to that observed in CA patients. All individuals underwent comprehensive two-dimensional (2D) Doppler echocardiography in conjunction with 3DSTE data acquisition during a single session. All measurements were performed by the same operator (AN). Detailed 3DSTE analysis was subsequently performed offline. The current analysis is a component of the MAGYAR-Path Study (Motion Analysis of the heart and Great vessels bY three-dimension Al speckle-tRacking echocardiography in Pathological cases). This cohort study aims to comprehensively analyze cardiac chamber and valvular functions across specific pathologies including CA (‘Magyar’ translates to ‘Hungarian’). This research was conducted with the approval of the Institutional and Regional Biomedical Research Committee (University of Szeged; Ref: 71/2011, latest approval 17 March 2025). All patients and control individuals provided informed consent.

### 2.2. Two-Dimensional Doppler Echocardiography

Standard transthoracic 2D Doppler echocardiographic examinations were performed to evaluate left atrial (LA) and LV dimensions, volumes, and LV ejection fraction (LV-EF) using Simpson’s biplane method. Diastolic function was assessed via Doppler-derived E/A ratios. Valvular pathologies, specifically regurgitation and stenosis, were evaluated and graded using color Doppler imaging and pressure gradient measurements [13]. All examinations were conducted using a Toshiba Artida™ ultrasound system (Toshiba Medical Systems, Tokyo, Japan) equipped with a 1–5 MHz PST-30BT phased-array transducer.

### 2.3. Three-Dimensional Speckle-Tracking Echocardiography

**Figure 1:**
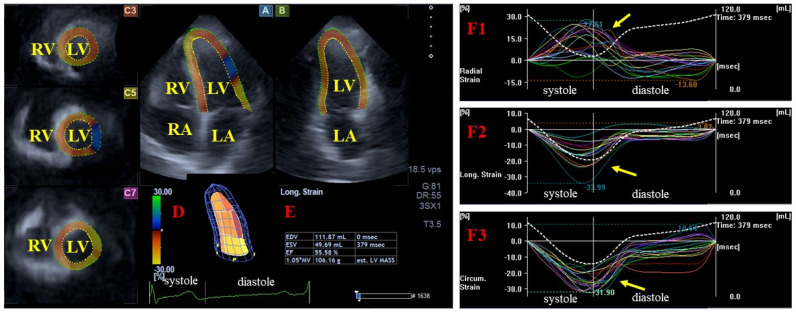
Assessment of left ventricular (LV) myocardial strains using three-dimensional (3D) speckle-tracking echocardiography. Panels (**A**) and (**B**) show the apical four-chamber and two-chamber long-axis views, respectively. Panels (**C3**,**C5**,**C7**) display short-axis views captured at the apical, mid-ventricular, and basal LV levels. (**D**) A virtual 3D LV cast illustrates the resulting model, with (**E**) presenting the derived LV volumes. Panels (**F1**,**F2**,**F3**) demonstrate global (white line) and segmental (colored lines) time–strain curves for radial, longitudinal, and circumferential deformation mechanics, respectively. On these panels, the corresponding time–LV volume change curves are also presented by dotted white lines. Yellow arrows represent the peak LV strains. Abbreviations: EDV, end-diastolic volume; EF, ejection fraction; ESV, end-systolic volume; LA, left atrium; LV, left ventricle, RA, right atrium; RV, right ventricle.

**Figure 2:**
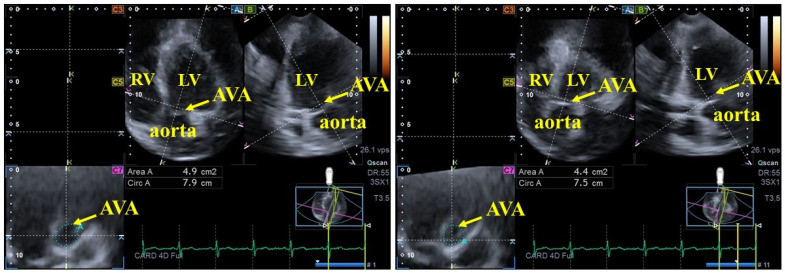
Evaluation of end-diastolic and end-systolic aortic valve annular dimensions and aortic valve annular spatial displacement, represented by aortic valve annular plane systolic excursion, via three-dimensional speckle-tracking echocardiography. Abbreviations: AVA, aortic valve annulus; Area A, AVA are; Circ A, AVA perimeter; LA, left atrium; LV, left ventricle; RV, right ventricle.

3DSTE studies were conducted in accordance with contemporary guidelines and established practices, using the same cardiac ultrasound equipment paired with a 3D-capable PST-25SX matrix-array transducer [9,10,11]. The 3DSTE procedure comprised two distinct phases:

- Data Acquisition: First, 3D echocardiographic datasets were acquired from the apical window. Following careful optimization of imaging parameters (e.g., gain, magnitude, etc.), six consecutive subvolumes were captured during six stable heart cycles while the patient maintained a breath-hold. The software subsequently stitched these subvolumes together to create a complete 3D volume.
- Data Analysis: Second, the acquired datasets were analyzed using the vendor-specific software, 3D Wall Motion Tracking (version 2.7, Toshiba Medical Systems, Tokyo, Japan).
  - For left ventricular (LV) strain analysis, the software automatically generated standard apical 4-chamber (AP4CH) and apical 2-chamber (AP2CH) long-axis views, along with three corresponding cross-sectional views. The observer manually defined the septal and lateral edges of the LV—mitral annulus and the endocardial surface of the LV apex. This initialization facilitated automated contour detection and sequential analysis, culminating in the creation of a virtual 3D LV model. The following basal regional unidirectional–unidimensional LV strains were subsequently measured: LV radial strain (RS, representing LV wall thickening/thinning), LV circumferential strain (CS, representing LV narrowing/widening) and LV longitudinal strain (LS, representing LV shortening/lengthening) (Figure 1) [9,10,11,14].
  - For AVA dimension measurements, optimal LV longitudinal planes were identified using the AP4CH and AP2CH views. The aortic valve and root were visualized by tilting and optimizing these planes until they were parallel to the aortic root centerline. A C7 cross-sectional view, aligned perpendicularly to the longitudinal plane, was used for measurement. Precise aligment was maintained to ensure the C7 plane remained perfectly perpendicular, while masurements specifically avoided the Valsalva sinuses and the LV outflow tract. The following AVA characteristics were measured during end-diastole (ED) and end-systole (ES): minimum and maximum AVA diameter (AVA-Dmin and AVA-Dmax, respectively), AVA area (AVA-A), measured by planimetry, and AVA perimeter (AVA-P), also measured by planimetry. Finally, AVA plane systolic excursion (AAPSE), defined as the spatial displacement of the AVA plane throughout the cardiac cycle, was also quantified (Figure 2) [9,10,11].

### 2.4. Statistical Analysis

All data are presented as mean ± standard deviation (SD) for continuous variables or as counts and percentages (n [%]) for categorical variables, as appropriate. Levene’s test was employed to assess the homogeneity of variances before further analysis. Depending on the data distribution and type, analyses were conducted using independent sample t-tests, analysis of variance (ANOVA), Fisher’s exact test, or the Kruskal–Wallis H test. A Bonferroni correction was applied to adjust for multiple comparisons. Pearson’s correlation coefficients were calculated between AAPSE and basal LV strains. The reproducibility of 3DSTE-derived AVA dimensions was evaluated by assessing intra- and interobserver variability in a cohort of 20 healthy subjects. Variability was quantified as the mean ± 2SD difference between repeated measurements (intraobserver) or measurements obtained by two independent observers (interobserver), accompanied by their respective interclass correlation coefficients (ICCs). Statistical significance was defined as a *p*-value < 0.05. All statistical computations were performed using SPSS software version 29.0.0.0 (SPSS Inc., Chicago, IL, USA).

## 3. Results

### 3.1. Clinical and Two-Dimensional Doppler Echocardiographic Data

In the CA group, 14 patients had hypertension, 7 patients had hypercholesterolaemia and 1 patient had diabetes mellitus. CA patients showed a larger LA diameter and thicker interventricular septum and LV posterior wall (Table 1). None of the CA patients and their matched controls had a history of atrial fibrillation. In the CA group, 19 had no aortic valve regurgitation (AR), while 4 had only mild AR. Similarly, 9 patients had no mitral regurgitation (MR), 8 patients had mild MR, and 6 patients had moderate MR. Mild, moderate and severe tricuspid regurgitation could be detected in 13, 6 and 4 CA patients, respectively. In the control group, only 1 subject had mild MR and 1 had mild TR; all others showed no MR or TR. None of the CA patients and controls showed significant valvular stenosis on any valves.

### 3.2. Three-Dimensional Speckle-Tracking Echocardiographic Data

In CA patients, AVA area was greater in end-diastole in 11 out of 23 cases (48%) and in end-systole in 8 out of 23 cases (35%), while it was equal in 4 out of 23 cases (17%). The ratio of healthy controls with greater end-diastolic AVA area (12 out of 23, 52%) and with greater end-systolic AVA area (11 out of 23, 48%) did not differ from that observed in CA patients.

End-diastolic and end-systolic maximum and minimum AVA diameters, areas and perimeters did not differ between CA patients and matched controls. AAPSE was found to be significantly impaired in all CA patients irrespective of AVA area size. Basal LV-RS, LV-CS and LV-LS were reduced in CA patients compared with controls, irrespective of AVA area size (except for basal LV-RS in the comparison between CA patients and healthy controls with greater end-diastolic AVA area). End-systolic AVA dimensions tended to be reduced in CA patients with greater end-diastolic AVA area as compared with those with greater end-systolic AVA area. Controls with greater end-diastolic AVA area showed lower basal LV-RS and LV-LS compared with those with greater end-systolic AVA area. While basal LV-RS and LV-CS proved to be similar between CA subgroups, basal LV-LS was tendentiously higher in CA patients with greater end-systolic AVA area (Table 2). CA patients with equal end-diastolic and end-systolic AVA area (*n* = 4) showed similarly reduced AAPSE (0.48 ± 0.08 cm), basal LV-RS (18.5 ± 7.3%), basal LV-CS (−20.1 ± 2.2%) and LV-LS (−12.0 ± 3.2%).

### 3.3. Correlation Analysis

AAPSE showed significant correlations with basal LV-RS (r = 0.692, *p* = 0.003) and LV-CS (r = −0.720, *p* = 0.002), but not with LV-LS (r = −0.331, *p* = 0.211).

### 3.4. Reproducibility of 3DSTE-Derived AVA Assessments

Table 3 presents the reproducibility of data for AVA assessments derived from 3DSTE. Specifically, it illustrates the mean ± 2SD difference for key parameters measured twice by the same examiner (intra-observer variability) and once by two independent examiners (inter-observer variability). The parameters evaluated include maximum and minimum AVA diameters, AVA area and AVA perimeter measured in end-diastole and end-systole. Furthermore, the table includes the respective ICCs to quantify the degree of agreement and consistency between these repeated measurements.

## 4. Discussion

Extracellular amyloid fibril infiltration leads to profound structural, morphological, and functional derangements of the cardiac chambers and valvular apparatus in CA [1,2,3]. Amyloid deposition within the AV leaflets may contribute to the pathophysiology of aortic stenosis. Augmented local inflammation and heightened mechanical stress are thought to play a pivotal role in the amyloidogenic process, promoting fibrillar accumulation within stenotic valves [15]. Although the role of AVA in LV function is essential, it has not been studied in CA. In recent research findings from the MAGYAR-Path Study, mitral and tricuspid annular dilation and functional impairment could be demonstrated in CA; however, similar investigations focusing on AVA have not yet been performed [1].

Cardiovascular imaging has experienced substantial development in recent decades. 3DSTE offers a notable advantage: it allows for the comprehensive, non-invasive assessment of all cardiac chambers, valves, and their annuli concurrently from a single acquired 3D echocardiographic dataset [16,17,18,19,20]. 3DSTE has been demonstrated for both LV strain and AVA assessments with well-defined normal references, enabling detailed (patho)physiological studies [9,10,11,21,22,23,24]. Despite established evidence confirming significant LV structural and functional abnormalities in CA patients [1], no prior studies have investigated the potential relationship between these findings and AVA dimensions or AAPSE measurements.

The fundamental function of the AV is to facilitate one-way flow from the LV to the aorta [25]. Contrary to the traditional view of a static structure, recent studies highlight that the AVA is dynamic: in healthy adults, the AVA area typically expands during systole, exceeding the end-diastolic area in nearly two-thirds of individuals examined [11,12]. Moreover, it exhibits a spatial displacement that is well represented by AAPSE. It was previously considered a representative of LV longitudinal function, as measured by M-mode echocardiography [26,27], but 3DSTE findings have shown that AAPSE has a more complex 3D movement rather than a single one-way displacement [9,10]. It has also been confirmed that healthy individuals exhibiting a larger end-diastolic AVA area display signs of subclinical LV dysfunction. These signs include impaired basal LV-RS, reduced LV-LS, and diminished basal rotation [28].

The findings of the present study suggest several important clinical implications. Primarily, it has been demonstrated that the comprehensive assessment of AVA dimensions, AAPSE (AVA plane systolic excursion), and regional LV strains can be achieved concurrently using a single methodology (3DSTE). This applies not only to healthy individuals but also to challenging cases such as those involving CA. However, there was a significant dropout due to poor image quality in CA group; hence, the technique seems to not be widely applicable. Second, all AVA dimensions in CA patients do not exhibit larger magnitudes compared with those of healthy individuals. Third, AVA area proved to be larger in end-diastole in 48%, in end-systole in 35%, and was equal in 17% of CA cases, which proved to be different from healthy cases according to the literature data (33%, 60% and 7%, respectively), which indicate a higher ratio of patients with greater end-diastolic AVA area or equal end-diastolic and end-systolic AVA area [28]. Fourth, attenuated AAPSE was observed within CA patients irrespective of the cardiac cycle phase in which AVA area are elevated. This phenomenon can be partly explained by the significant reduction in all LV strains. According to the literature data, AAPSE impairment can be observed in noncompaction cardiomyopathy, together with reduced basal LV strains and the frequent presence of greater end-diastolic AVA area, which are not associated with AVA dilation like in CA [9]. In acromegalic cardiomyopathy, impaired AAPSE and dilated AVA were associated with preserved basal LV strains and equal frequency of greater end-diastolic and end-systolic AVA area. However, differences in parameters according to disease activity could be detected [10]. These findings suggest that AAPSE could serve as a marker of disease severity or progression; therefore, follow-up studies are warranted to assess its prognostic value. All the findings presented in this study suggest significant abnormalities in AVA dynamics in CA. However, further large-scale studies employing more accurate and reliable quantification methods are necessary to validate and confirm the findings presented here. Hopefully, future technological developments will allow for the more accurate—even automated—consideration and measurement of the spatial displacement of AVA, as represented by AAPSE, making this analysis a possible new diagnostic and prognostic method.

## 5. Limitations

Several limitations impact the generalizability of our findings:

- 3DSTE currently offers lower spatial and temporal resolutions compared to 2D echocardiography. Furthermore, the larger transducer size poses challenges for optimal chest positioning, and the multi-cycle acquisition process needed for 3D reconstruction may induce stitching or motion artifacts [16,17,18,19,20].
- The study population size for CA patients (*n* = 35) was limited by the rarity of the disease and the single-center design, restricting statistical power [1,2,3,4,5,6,7,8]. Therefore, it is important to emphasize that the presented findings are preliminary and need to be validated in larger, multi-center cohorts. Moreover, adding a multivariate analysis to adjust for key confounders (e.g., hypertension, LV wall thickness) in future work will be important.
- The high prevalence of certain cardiovascular risk factors among CA patients may act as potential confounders influencing the results. However, these facts are known features of CA.
- The scope of the analysis was intentionally constrained to specific AVA parameters, excluding other available STE-derived parameters.
- Valvular regurgitations were assessed qualitatively (visually), rather than using more advanced, quantitative scoring systems [29]. Therefore, the use of quantitative Doppler or 3D-based echocardiographic methods is recommended for future studies.

## 6. Conclusions

In the presence of CA, AVA is not dilated but its spatial displacement is reduced, suggesting its functional impairment, as represented by AAPSE, possibly due to the reduction in all concomitant LV strains, suggesting significantly abnormal AVA dynamics in CA.

## Figures and Tables

**Table 1:** Two-dimensional echocardiographic data of patients with cardiac amyloidosis and controls.

	Controls (*n* = 23)	All CA Patients (*n* = 23)
LA diameter (mm)	39.1 ± 5.1	46.3 ± 6.5 *
LV end-diastolic diameter (mm)	47.4 ± 3.8	47.7 ± 4.7
LV end-diastolic volume (mL)	105.8 ± 21.1	115.9 ± 27.5
LV end-systolic diameter (mm)	31.1 ± 3.3	30.8 ± 4.5
LV end-systolic volume (mL)	36.0 ± 9.3	44.6 ± 13.0
Interventricular septum (mm)	9.6 ± 1.4	14.3 ± 1.8 *
LV posterior wall (mm)	9.8 ± 1.5	13.6 ± 1.4 *
LV ejection fraction (%)	65.6 ± 3.5	59.8 ± 11.9
E (cm/s)	71.2 ± 16.4	81.5 ± 20.5
A (cm/s)	73.4 ± 18.8	56.9 ± 30.2

Abbreviations: CA = cardiac amyloidosis; LA = left atrial; LV = left ventricular. * *p* < 0.05 vs. Controls.

**Table 2:** Comparison of three-dimensional speckle-tracking echocardiography-derived aortic valve annular dimensions and aortic valve plane systolic excursion between patients with cardiac amyloidosis and controls.

	All Controls (*n* = 23)	Controls with Greater End-Diastolic AVA-A (*n* = 12)	Controls with Greater End-Systolic AVA-A (*n* = 11)	All CA Patients (*n* = 23)	CA Patients with Greater End-Diastolic AVA-A (*n* = 11)	CA Patients with Greater End-Systolic AVA-A (*n* = 8)
AVA-Dmax-D (cm)	2.05 ± 0.26	2.10 ± 0.26	1.99 ± 0.24	2.10 ± 0.48	2.11 ± 0.35	2.11 ± 0.19
AVA-Dmin-D (cm)	1.86 ± 0.26	1.92 ± 0.22	1.80 ± 0.28	1.80 ± 0.34	1.73 ± 0.24 ‡	1.95 ± 0.40
AVA-A-D (cm^2^)	3.27 ± 0.76	3.44 ± 0.79	3.08 ± 0.68	3.27 ± 0.72	3.34 ± 0.73	3.20 ± 0.61 *
AVA-P-D (cm)	6.46 ± 0.76	6.63 ± 0.79	6.27 ± 0.67	6.47 ± 0.73	6.46 ± 0.77	6.54 ± 0.61
AVA-Dmax-S (cm)	1.96 ± 0.29	1.91 ± 0.28	2.02 ± 0.28	1.99 ± 0.39	1.87 ± 0.34	2.16 ± 0.34
AVA-Dmin-S (cm)	1.90 ± 0.27	1.84 ± 0.27	1.97 ± 0.25	1.74 ± 0.29	1.67 ± 0.28	1.86 ± 0.23
AVA-A-S (cm^2^)	3.19 ± 0.85	2.88 ± 0.83 #	3.54 ± 0.73	3.28 ± 0.83	3.03 ± 0.70	3.66 ± 0.83
AVA-P-S (cm)	6.33 ± 0.90	6.04 ± 0.90	6.65 ± 0.79	6.46 ± 0.84	5.21 ± 0.77	6.86 ± 0.76
AAPSE (cm)	1.13 ± 0.23	1.06 ± 0.20	1.20 ± 0.24	0.60 ± 0.26 †	0.66 ± 0.31 ‡	0.56 ± 0.20 @
basal LV-RS (%)	33.7 ± 12.0	27.6 ± 8.7 #	39.8 ± 11.7	19.6 ± 8.4 †	21.0 ± 10.3	20.0 ± 6.3 @
basal LV-CS (%)	−27.2 ± 6.0	−28.0 ± 6.4	−26.5 ± 5.5	−19.9 ± 7.2 †	−20.5 ± 9.1 ‡	−19.2 ± 5.8 @
basal LV-LS (%)	−21.5 ± 4.6	−19.1 ± 3.4 #	−23.9 ± 4.3	−13.3 ± 5.4 †	−11.6 ± 5.3 ‡	−15.5 ± 5.2 @

Abbreviations: CA = cardiac amyloidosis; AVA = aortic valve annulus; Dmax = maximum diameter; Dmin = minimum diameter; A = area; P = perimeter; D = end-diastolic; S = end-systolic; AAPSE = aortic valve annulus plane systolic excursion. * *p* < 0.05 vs. end-systolic counterpart; † *p* < 0.05 vs. all Controls; ‡ *p* < 0.05 vs. Controls with greater end-diastolic AVA-A; # *p* < 0.05 vs. controls with greater end-systolic AVA-A; @ *p* < 0.05 vs. Controls with greater end-systolic AVA-A.

**Table 3:** Intra- and interobserver variability for three-dimensional speckle-tracking echocardiography-derived assessment of aortic valve annular dimensions and aortic valve plane systolic excursion.

	Intraobserver Agreement		Interobserver Agreement	
	Mean ± 2SD Difference in Values Obtained by 2 Measurements of the Same Observer	Correlation Coefficient Between Measurements of the Same Observer	Mean ± 2SD Difference in Values Obtained by 2 Observers	Correlation Coefficient Between Independent Measurements of 2 Observers
AVA-Dmax-D (cm)	−0.05 ± 0.21	0.88 (*p* < 0.01)	−0.08 ± 0.16	0.887 (*p* < 0.01)
AVA-Dmin-D (cm)	−0.02 ± 0.18	0.91 (*p* < 0.01)	−0.03 ± 0.21	0.92 (*p* < 0.01)
AVA-A-D (cm^2^)	−0.11 ± 0.55	0.93 (*p* < 0.01)	−0.13 ± 0.56	0.94 (*p* < 0.01)
AVA-P-D (cm)	−0.06 ± 0.59	0.92 (*p* < 0.01)	−0.13 ± 0.69	0.94 (*p* < 0.01)
AVA-Dmax-S (cm)	0.02 ± 0.35	0.91 (*p* < 0.01)	0.04 ± 0.31	0.94 (*p* < 0.01)
AVA-Dmin-S (cm)	0.07 ± 0.28	0.83 (*p* < 0.01)	0.02 ± 0.29	0.80 (*p* < 0.01)
AVA-A-S (cm^2^)	0.12 ± 0.54	0.91 (*p* < 0.01)	0.10 ± 0.69	0.95 (*p* < 0.01)
AVA-P-S (cm)	−0.04 ± 0.51	0.92 (*p* < 0.01)	0.03 ± 0.49	0.93 (*p* < 0.01)
AAPSE (cm)	−0.04 ± 0.18	0.90 (*p* < 0.01)	−0.03 ± 0.25	0.93 (*p* < 0.01)

Abbreviations: AVA = aortic valve annulus; Dmax = maximum diameter; Dmin = minimum diameter; A = area; P = perimeter; D = end-diastolic; S = end-systolic; AAPSE = aortic valve annulus plane systolic excursion.

## Data Availability

The data presented in this study is available on request from the corresponding author.
